# Complete chloroplast genomes and phylogenetic relationships of *Pedicularis chinensis* and *Pedicularis kansuensis*

**DOI:** 10.1038/s41598-024-63815-0

**Published:** 2024-06-21

**Authors:** Tao Wang, Xiuzhang Li, Chuyu Tang, Zhengfei Cao, Hui He, Xiaoping Ma, Yuling Li, Kejia De

**Affiliations:** 1 State Key Laboratory of Plateau Ecology and Agriculture, Qinghai University, Xining, 810016 China; 2 Qinghai Academy of Animal and Veterinary Science, Xining, 810016 China; 3 Menyuan Hui Autonomous County Grassland Station, Menyuan, 810300 China

**Keywords:** Phylogenetics, Taxonomy

## Abstract

The complete cp genomes of *Pedicularis chinensis* (GenBank accession number: OQ587614) and *Pedicularis kansuensis* (GenBank accession number: OQ587613) were sequenced, assembled, and annotated. Their chloroplast (cp) genome lengths were 146,452 bp, and 146,852 bp, respectively; 120 and 116 genes were identified, comprising 75 and 72 protein-coding genes (PCGs), 37 and 36 transfer RNA (tRNA) genes, and 8 and 8 ribosomal RNA (rRNA) genes, for *P. chinensis* and *P. kansuensis*, respectively. A simple sequence repeat (SSR) analysis revealed that the repetitive sequences were mainly composed of mononucleotide repeats (A/T motif) and dinucleotide repeats (AT/TA motif). Comparative genomics identified several variant genes (*rpl22*, *rps19*, *rpl12*, *ycf1*, *trnH*, *psbA*, and *ndhH*) and variant regions (*trnS*-*GGA*, *trnV*-*UAC*, *ndhJ*-*trnV*, *ycf4*-*cemA*, *ndhE*-*nhdG*, and *rpl32*-*trnL*) with a high Pi, indicating the potential to serve as deoxyribo nucleic acid (DNA) barcodes for *Pedicularis* species identification. The results show that the cp genomes of *P. chinensis* and *P. kansuensis* were the same as those of other plants in *Pedicularis*, with different degrees of AT preference for codons. Large differences in the number of SSRs and the expansion of the inverted repeat (IR) region showed strong variability and interspecific differentiation between these two species and other species represented in the genus *Pedicularis*. A phylogenetic analysis showed that *P. kansuensis* had the closest relationship with *P. oliveriana*, and *P. chinensis* had the closest relationship with *P. aschistorhyncha*. These results will facilitate the study of the phylogenetic classification and interspecific evolution of *Pedicularis* plants.

## Introduction

*Pedicularis* Linn. 1753 plants are strongly differentiated, with complex morphological variation, abundant and diverse habitats, and different centers of species origin due to the dual effects of their natural reproductive isolation and their rapid radial differentiation and reproduction mode^1,2^, resulting in a rich and diverse genus. On the one hand, they provide extremely rich germplasm resources for genetic breeding of the genus; on the other hand, they provide great difficulties for taxonomic and systematic studies of the genus. Globally, there are about 568 recognized species, 335 synonyms, and 450 unidentified species^3^. Asia has the highest biodiversity, with about 350 species in China, of which 271 species are endemic; 83 species in India; and 70 species in Europe^4–6^. Chloroplast is an organelle with an advanced autonomous genetic system and a complete genome. The cp genome of angiosperms has a circular tetrad structure, which is a large single-copy region (LSC), a small single-copy region (SSC) and a pair of separate inverted repeat regions (IRs)^7,8^. The chloroplast genome has the characteristics of short sequence (120–160kb), containing conserved sequence regions (*matK*, *rbcL*, *trnH*-*psbA* and *trnL*-*F*), rich simple repeat sequence sites, easy extraction and purification, and parthenogenetic inheritance^9^. Compared with the nuclear genome, its genome composition and gene type are more conservative and genetic stability, the evolution rate is moderate, and there is no gene recombination^10^. The cp genome sequence contains more genetic information than a single gene^11^, in which simple repeat sequences can be used as effective molecular markers to detect population polymorphism, and are widely used in molecular assisted breeding and species protection^12^. Codons are the link between nucleic acids and proteins and play an important role in the transmission of genetic information^13^. It is widely used at different levels of plant research, such as molecular identification, phylogeny, population evolution, and plant origin^14^. In recent years, the cost of cp genome sequencing has been greatly reduced, and more and more cp genome data have been successfully applied to plant phylogenetic and evolutionary studies^15^. Among them, the most prominent is to define the phylogenetic status and genetic diversity of the new species by measuring the cp genome of the new species and conducting comparative analysis^16^.

It is generally recognized that the cp genome structure and gene content are relatively conserved, but the cp genome sequence of *Pedicularis* has undergone a strong IR expansion, resulting in the loss of sequence regions^17,18^. In the cp genomes of *P. verticillata* and *P. cheilanthifolia*, gene loss is not a special phenomenon, such as the loss of *ndh* genes (*ndhA* and *ndhK*), and the *rps16* gene was lost only in *P. verticillata*^19–21^. A reduction in the cp genome length due to the loss of *ndh* has also been observed in *P. hallaisanensis* and *P. alaschanica* in the last 5 years^22^. Similar reports have also indicated that the chloroplast genome of this genus is highly variable, with gene rearrangements occurring in a few species^23,24^. This implies that the variable morphological features of the genus may be somehow associated with sequence variation in the cp genome. As more data on the cp genome of *Pedicularis* have been reported, with the help of single-nucleotide polypeptide (SNP), short tandem repeats (STRs), and genetic polymorphism^25,26^, scholars have been prompted to examine the relationship between diversity and cp genome sequence variation in this genus.

Therefore, the determination of cp genome data for more species of the genus is necessary. In this study, the complete cp genomes of *P. chinensis* Maxim. and *P. kansuensis* Maxim. were determined, annotated, and compared with the cp genomes of reported *Pedicularis* plants. Therefore, the phylogenetic status of the two new species was determined, and the genetic data of the species of the genus *Pedicularis* were enriched.

## Results

### Comparative analysis of chloroplast genome structure of *Pedicularis*

**Figure 1:**
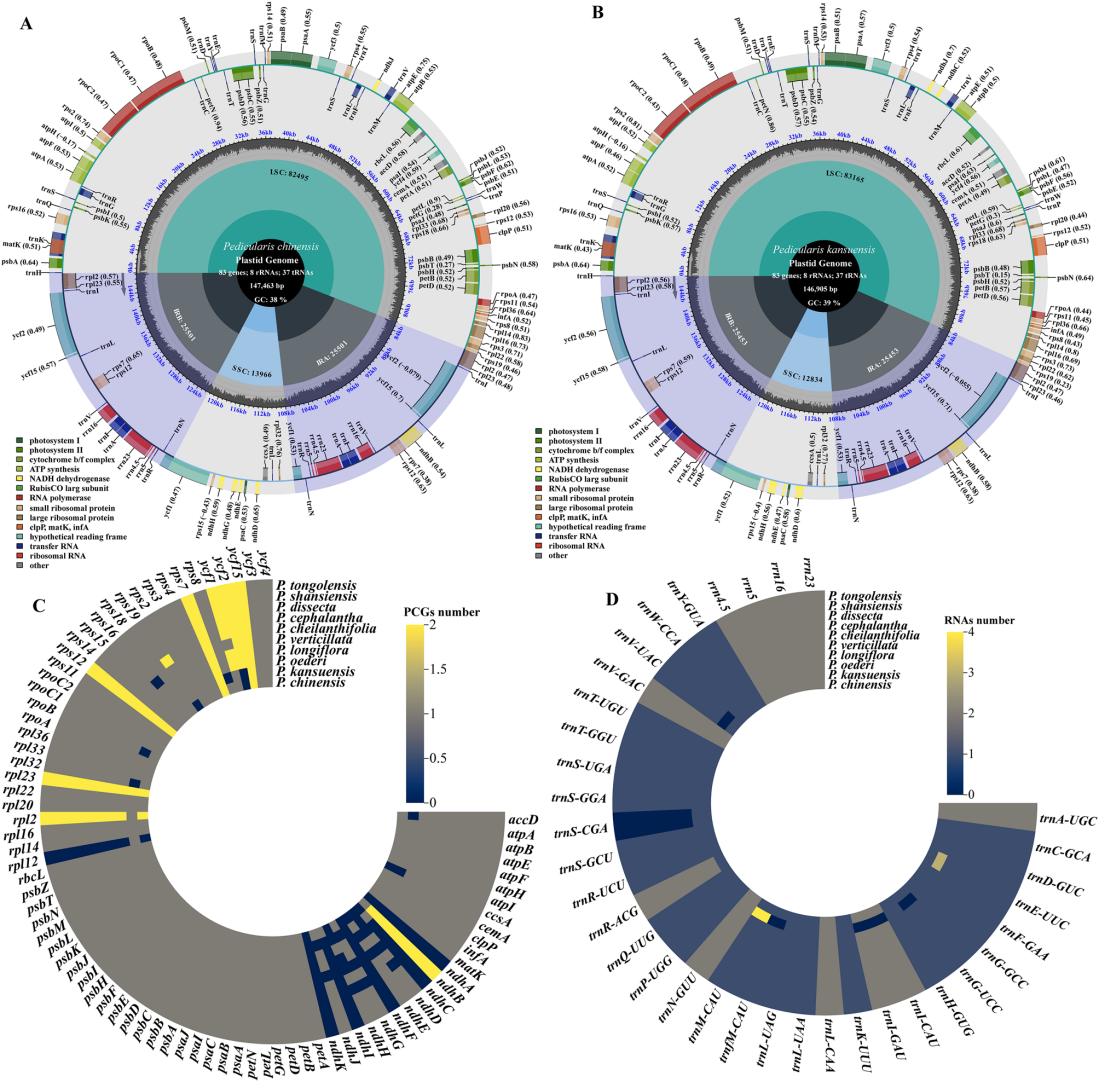
Chloroplast genome structure of *Pedicularis*. (**A**) The *P. chinensis* cp genome map. (**B**)The *P. kansuensis* cp genome map. The genes inside the circles are transcribed clockwise, and those outside are transcribed counterclockwise. The dark gray in the inner circle shows the G + C content, while the light gray shows the A + T content. The legend identifies genes with different functions. The distribution of PCGs (**C**) and RNAs (**D**) in the cp genomes is shown. (**C**) and (**D**) represent the distribution number of protein-coding genes and RNA-coding genes in plant cp genome, respectively. Brighter colors (yellow) correspond to more of the gene being contained.

The whole cp genomes of *P. chinensis* and *P. kansuensis* were assembled and spliced into a covalently closed-loop tetrameric structure with total sequence lengths of 146, 452 bp and 146, 852 bp, respectively, consisting of an LSC (82, 518 bp and 83, 190 bp), an SSC (13, 836 bp and 12, 834 bp), and a pair of inverted repeat regions (IR) (25, 049 bp and 25, 414 bp) (Fig. 1A,B).

The complete cp genome of *P. chinensis* encoded a total of 120 genes, comprising 75 PCGs (protein-coding genes), 37 transfer RNA (tRNA) genes, and 8 ribosomal RNA (rRNA) genes. The LSC region contained 60 PCGs and 22 tRNAs; the SSC regions contained 5 PCGs and 1 tRNA; and the IR regions contained 10 PCGs, 14 tRNAs, and all rRNA genes. The *P. kansuensis* cp genome encoded 116 genes, comprising 72 protein-coding genes, 36 tRNA genes, and 8 rRNA genes. The LSC region contained 58 PCGs and 21 tRNA; the SSC regions contained 4 PCGs and 1 tRNA; and the IR regions contained 10 PCGs, 14 tRNA, and all rRNA genes (Table S1). At present, the published cp genomes of 15 *Pedicularis* species encode 102-109 genes, 69-74 PCG genes, 30 tRNA genes, and 8 rRNA genes. The cp of *P. kansuensis* and *P. chinensis* are similar to those of other *Pedicularis* plants in terms of structure, gene content, and gene sequencing, and no obvious gene rearrangement or inversion was found. However, some genes underwent specific changes, for example, deletions and multiple copies.

In a comparative analysis of 10 *Pedicularis* species, it was found that the *rps19* gene was pseudogenized across the LSC/IRb boundary. Gene loss seems to be a general phenomenon during the evolution of *Pedicularis* plants, with *P. chinensis* and *P. kansuensis* losing two PCGs genes, *ccsA* and *ycf2* (Fig. 1C), while encoding more tRNAs (*trnE*-*UUC*, *trnM*-*CAU,* and *trnS*-*CGA*) (Fig. 1D); in contrast, genes such as *ndhD*, *ndhJ*, and *ndhH* showed different levels of deletion among species.

### PCGs codon usage analysis

**Figure 2:**
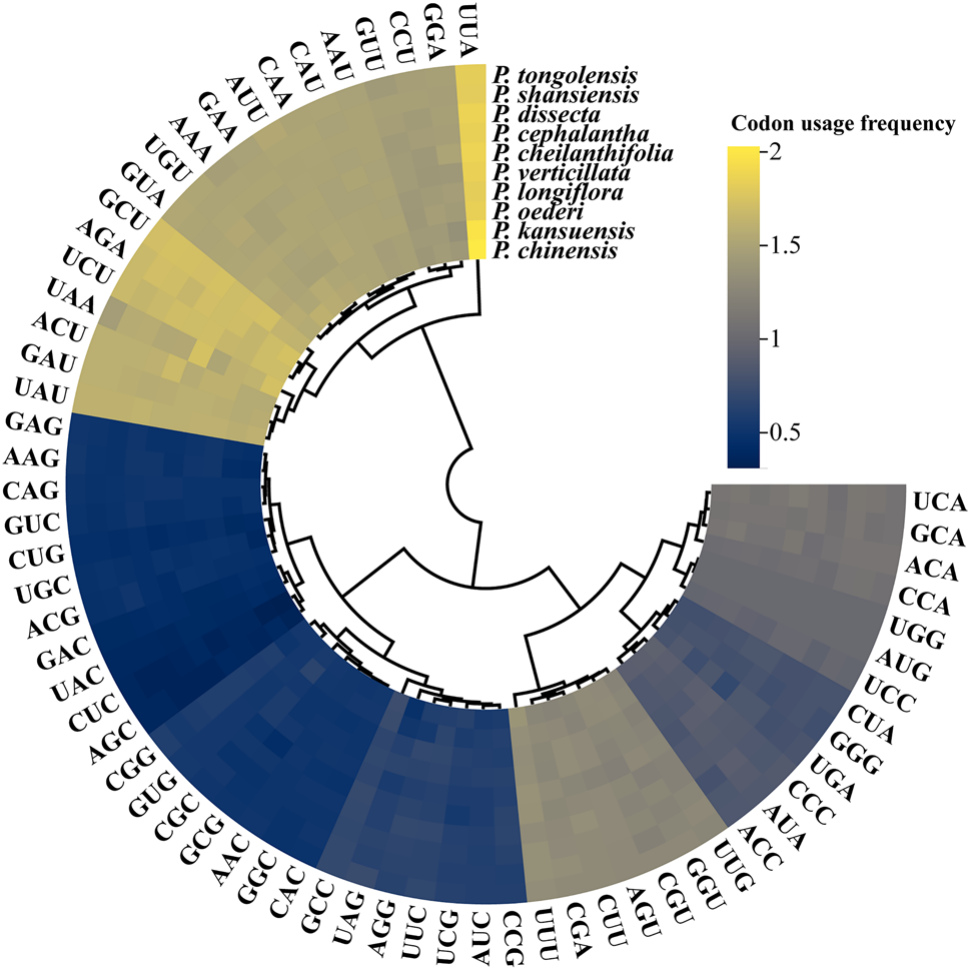
Distribution of synonymous codon usage (relative synonymous codon usage, RSCU) for 10 species of *Pedicularis*. Higher values indicated in yellow correspond to higher RSCU values, and blue values correspond to similar values.

The results show that there was no preference for tryptophan (Trp) (UGG) or methionine (Met) (AUG) in *Pedicularis* plants (RSCU = 1) (Fig. 2). The usage of leucine (Leu) had the highest value of RSCU (2.03) for UUA, and the use of CUC was the lowest (0.31). This indicates that the UUA codon is the most preferred for Leu in *Pedicularis* plants, while the CUC codon is less selected by *Pedicularis* plants. Among the codons with higher RSCU values, such as UAU, GAU, ACU, and GCU, the A + T content was higher and showed A + T bias. The similarity in the codon usage values found for *P. chinensis* and *P. kansuensis* is also consistent with the preference for codons observed for other *Pedicularis* species. To better analyze the cp genome codon preference in *Pedicularis* plants, the use of cp genome codons in 10 plant species was examined for a comparative analysis. In a visualized heat map of codon distribution used for the 10 selected species, it is shown that about one-third of the codons are not commonly used (Fig. 2).

### Analyses of repeat types and simple sequence repeats

**Figure 3:**
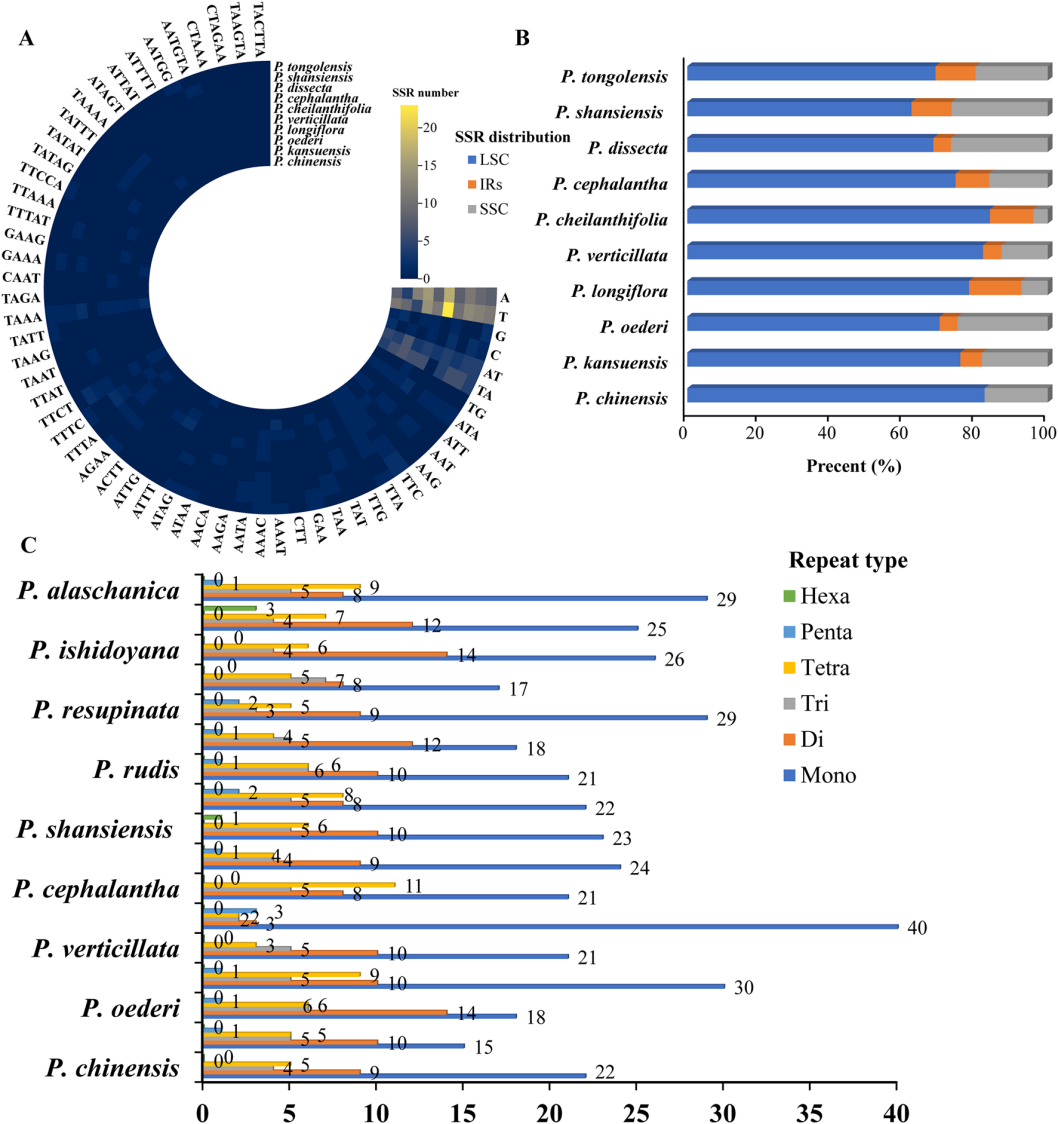
SSRs analysis of *Pedicularis* species. (**A**) Show the repeat motif of identified SSRs. (**B**) Show the distribution of different SSR types in the plastome regions. (**C**) Show the abundance of different SSR types.

In the cp genome of *Pedicularis* plants, mononucleotide repeats (A/T motif) and dinucleotide repeats (AT/TA motif) were found to be the most abundant, confirming the high A+T bias in the *Pedicularis* species (Fig. 3A). In the cp genomes of *P. kansuensis* and *P. chinensis*, 40 and 36 SSRs were found, respectively, slightly lower than in other *Pedicularis* species (37–55 SSRs). Furthermore, most *Pedicularis* species did not have hexanucleotide repeats, such as CTAGAA, TAAGTA, and TACTTA (Fig. 3A), which was the only cp genome that had this repeat type. This suggests that the loss of long-nucleotide repeats in *P. kansuensis* and *P. chinensis* is a common feature during the evolution of *Pedicularis*.

The SSRs of *Pedicularis* plants differed greatly in their distribution preferences in different regions of the cp genome, mostly in the SSC and LSC regions, and less or not at all in different cp genome regions, as they were found mostly in the SSC and LSC regions. Among them, the SSRs of *P. chinensis* were predominantly distributed in the LSC region (82.50%), while no SSRs were observed in the IR region, which may imply that the IR region of *P. chinensis* is not highly variable and does not have potential as a source of molecular markers such as SSRs. The distribution of SSRs among the other plants was not strong, although it did also vary (Fig. 3B). Comparing the type and number of SSRs in *Pedicularis* species, it was found that all had four types, namely, mono-, di-, tri, and tetra-nucleotide repeats, but the type and tetranucleotide repeat content units varied considerably (Fig. 3A and C). The results show that, in the process of *Pedicularis* plant evolution, the changes in SSR richness and species among individuals greatly promote the improvement of species genetic diversity, and the LSC and SSC regions are regions of high mutation.

### Analysis of sequence variation

**Figure 4:**
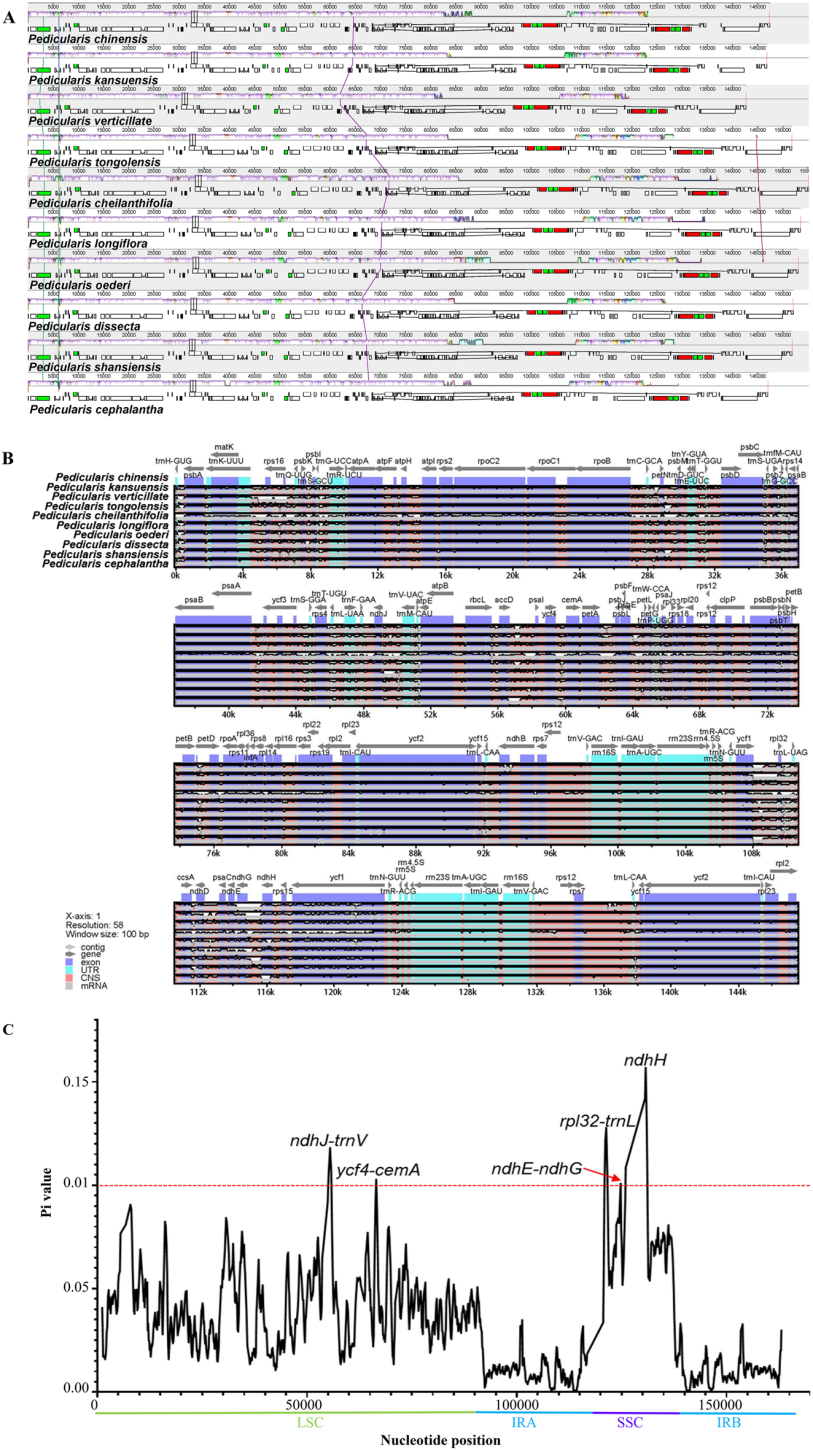
Analysis of sequence variability of *Pedicularis*. (**A**) The comparison of cp genome covariance of *Pedicularis*. Identical color blocks indicate homologous regions, and homologous blocks are connected using line segments to reflect the trend of fragment similarity; white rectangles are PCGs, red rectangles are rRNAs, green rectangles are tRNAs. (**B**) Sequence variability characteristics of 10 species of *Pedicularis*. (**C**) The cp genome nucleotide diversity of *Pedicularis*.

To compare the arrangement of homologous genes or sequences in *Pedicularis* plants, evaluate the retention or loss of homologous genes in cp genomes, and finally analyze the evolutionary relationship of *Pedicularis*. It was found that the cp genomes of *Pedicularis* species had good collinearity, and no gene rearrangement or inversions were found in the cp genomes of *Pedicularis* species (Fig. 4A), which indicates their conservative character. *P. kansuensis* and *P. chinensis* showed no significant differences in genome composition or size among the studied cp genomes, with a high sequence similarity, few variation sites, and small interspecific differences (Fig. 4B). At the same time, it was found that the coding region was more conservative than the non-coding region, the IR region was more conservative than the LSC and SSC regions, and the SSC region was the variation hot spot. The sequences of the genes *rpl22*, *rps19*, *rpl12*, *ycf1*, *trnH*, *psbA*, and *ndhH* and the non-coding regions of *trnS*-*GGA*, *trnV*-*UAC*, *ndhJ*-*trnV*, *ycf4*-*cemA*, *ndhE*-*nhdG*, and *rpl32*-*trnL* were characterized by the highest nucleotide diversity. To measure the degree of genetic variation in *Pedicularis*, genetic variations in species and related species were analyzed to determine evolutionary relationships. We analyzed and compared the cp genome sequence diversity of 10 *Pedicularis* species, including *P. kansuensis* and *P. chinensis,* and they are expressed as nucleotide variability values (Pi). It was found that Pi ranged from 0 to 0.15671 in the 10 *Pedicularis* species, indicating a high degree of sequence variation among the studied species (Fig. 4C). The four high-mutation regions (Pi > 0.1) detected in the study, *ndhJ*-*trnV*, *ycf4*-*cemA*, *ndhE*-*nhdG,* and *rpl32*-*trnL,* and one high-mutation gene (*ndhH*) were distributed in the LSC and SSC regions, with the lowest Pi values in the IR region, higher Pi values in the LSC region, and the highest Pi values in the SSC region. This indicates that, compared to the IR region, the LSC and SSC regions are high-mutation regions with a higher nucleotide diversity and that the SSC region has the strongest variability, agreeing with the results of the sequence variation analysis. This may be related to the large number of repetitive sequences in the LSC and SSC regions, where multiple duplications of variant sites in the same region increase variability. The reverse duplication of genes in the IR region resulting in a conserved region also implicitly supports this conclusion. Five highly divergent regions were found in the intergenic region; this suggests that the intergenic region is more variable than the coding region, indicating that the *Pedicularis* plant genome has a rich polymorphism. In this study, the nucleotide variation of *ndhH* located in the SSC region was the largest (Pi = 0.15671), indicating that the SSC region is a mutation active region, and its highly variable sequence may be used as a candidate DNA barcode for this species. Artificially verifying the application of these markers in *Pedicularis* plants will be a very meaningful work, because it can analyze the phylogenetic status of *Pedicularis* plants by providing key genomic evidence.

### IR contraction and expansion

**Figure 5:**
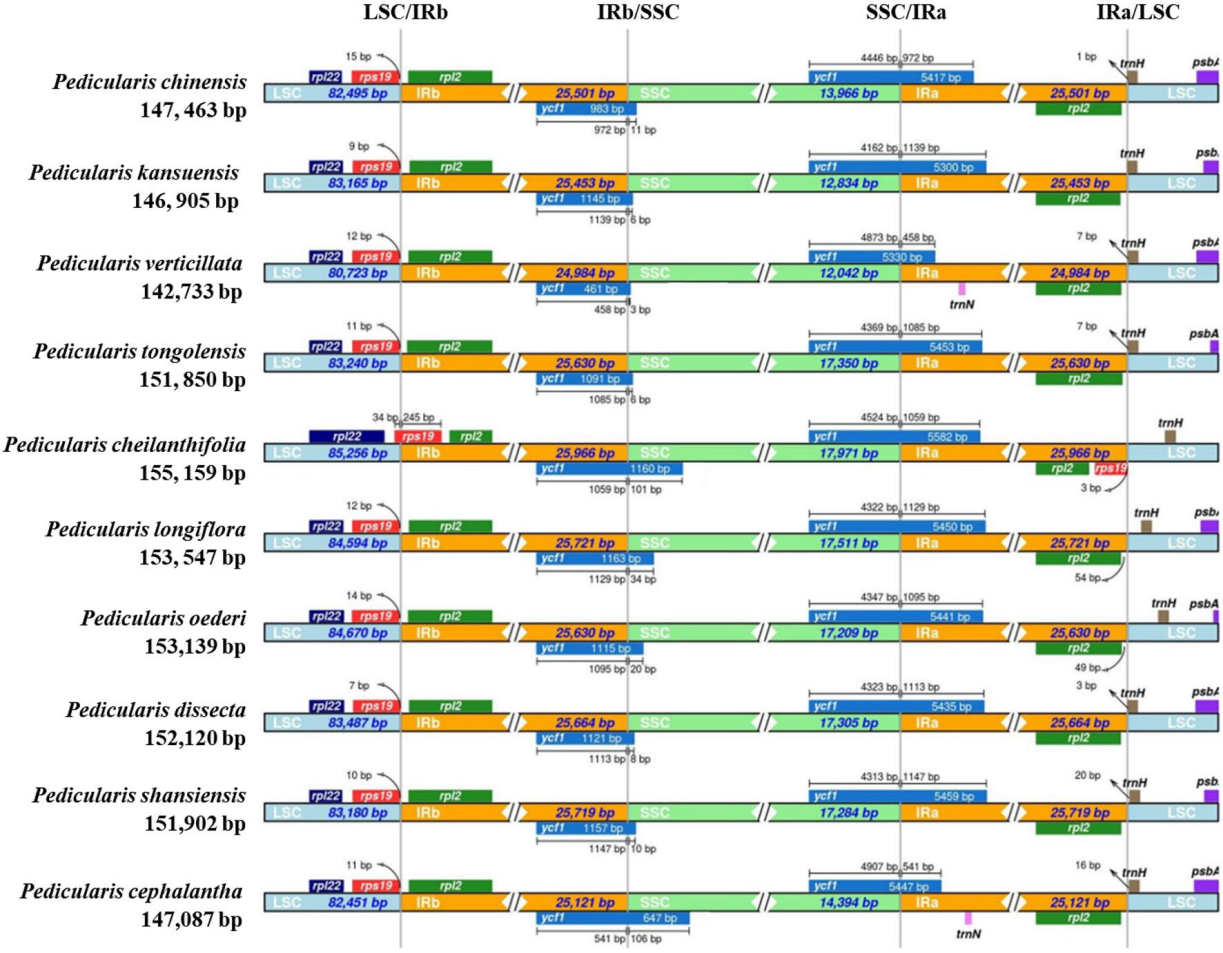
Expansion and contraction of different regions of cp in *Pedicularis*. The color box above the horizontal line represents the genes and gene fragments that cross the boundary, and the number represents the distance between the gene boundary (the starting point or end point of the gene) and the connection site. The fragments and genes in the LSC, IR and SSC regions were not arranged in proportion.

The comparative analysis of the cp genomes of 10 *Pedicularis* species revealed the longest IR region in *P. cheilanthifolia* (25,966 bp) and the shortest in *P. verticillata* (24,984 bp), which yielded a difference of 982 bp, and the IR region of *P. cheilanthifolia* was 3.94% longer than that of *P. verticillata* (Table S1). It was found that there was a significant expansion of the IR region in the 10 *Pedicularis* species. The *rps19* gene crossed the LSC/IRb boundary, the *ycf1* gene crossed the IRb/SSC and SSC/IRa boundaries due to its reverse repeat in the IR region, and *trnH* crossed the IRa/LSC boundary (Fig. 5).

In the 10 *Pedicularis* species, most of the *rps19* gene sequences were located within the LSC region, with 7–15 bp extending into the IRb region. However, only 34 bp (12.18%) of the *rps19* gene of *P. cheilanthifolia* remained within the LSC region, and 245 bp (87.82%) of the gene extended into the IRb region, indicating that the expansion of the IR region of *P. cheilanthifolia* was more drastic, nearly annexing the entire *rps19* gene. The *trnH* genes were affected by the expansion of the IR region and varied in distance from the IRA/SSC boundary (0–20 bp). An interesting phenomenon is that only 3-106 bp of the *ycf1* gene remained within the SSC region when crossing the SSC/IRb boundary, with most of the genes being within the IRb region. In contrast, most of the gene was within the IRa region when crossing the SSC/IRa boundary, with 458–1149 bp of the *ycfl* pseudogene sequence extending into the IRa region. However, considering that the *ycf1* gene repeats twice, which is characteristic of composite IR region genes, it is more likely to be located in the IR region, whereas it differs significantly in gene length when crossing the IRb/SSC boundary and the SSC/IRa boundary. This indicates that the *ycf1* gene is affected by the expansion of the IR region to different degrees when crossing the SSC/IRb and SSC/IRa boundaries, resulting in the gene fragments that account for the majority of its gene sequences being attributed to different regions. Thus, the expansion of the IR region in *Pedicularis* leading to the variability of *rps19* and *ycf1* genes will greatly facilitate researchers' insight into the evolution of the cp genome of *Pedicularis* species.

### Phylogenomic analysis

**Figure 6:**
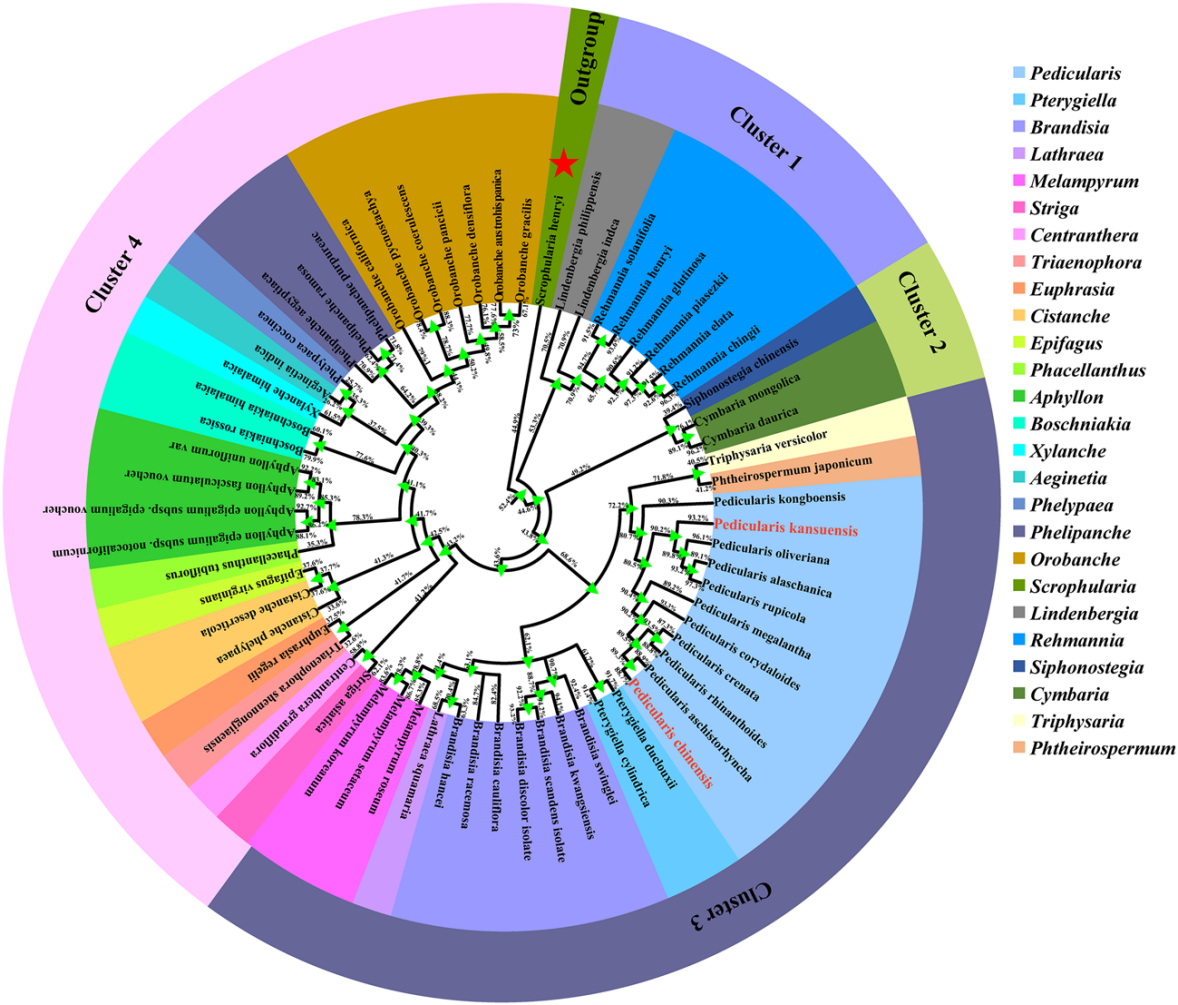
Phylogenetic analysis of the cp genomes of *Pedicularis* species and selected representatives of the family Orobanchaceae based on the maximum likelihood method (ML). In the figure, the green triangles represent the nodes, and the numbers are bootstrap (BP) support values. The species whose cp genomes are reported in this study are marked with red font. The red star species is S*crophularia henryi* (outgroup). The legend on the right shows all genera.

There is a clear evolutionary relationship between 66 species in 25 genera of Orobanchaceae, which can be divided into 3 clusters. *Pedicularis*, *Phtheirospermum*, *Triphysaria*, *Pterygiella*, *Brandisia*, *Lathraea* and *Melampyrum* were clustered 3. Among them, *Pedicularis* was clustered with *Phtheirospermum* and *Triphysari*a (BP = 72.2%), and had a close interspecific relationship. The maximum likelihood (ML) method of phylogenetic analysis supported that *P. kansuensis* and *P. olivriana* (BP = 89.3%), *P. chinensis* and *P. aschistorhyncha* (BP = 90.2%) had the closest sister relationship (Fig. 6).

## Discussion

In this study, the cp genomes of *P. kansuensis* and *P. chinensis* were reported and analyzed in comparison with other *Pedicularis* plants for the first time. As with traditional terrestrial plants, the cp genomes of *Pedicularis* plants comprise LSC, SSC, and IR regions and exhibit a typical cp genome structure^27^. The number and order of genes encoded in the cp genomes of both species did not differ from the plastomes of other *Pedicularis* species, while the types and numbers of genes lost during evolution differed. A comprehensive comparison revealed that the LSC region of the cp genome was more variable and had more active evolutionary behavior, probably because it contains fewer conserved genes and conserved regions^28^. We also found that both conserved genes were distributed in the IR regions, and the conserved sequences were distributed in the IR region, which directly led to the IR region being the most conserved and the SSC region becoming a hotspot region for structural variation in the cp genome.

It has been suggested that a lost gene may be pseudogenized, and when it is considered a pseudogene, it loses the ability to fully replicate itself and is no longer able to encode a complete protein, resulting in the loss of biological function^29,30^. It has also been reported that, when a gene becomes pseudogenized, it is only the original functional gene that is inactivated by nucleotide deletions, mutations, and inversions, resulting in the loss of the functional gene^31,32^. However, it can still encode proteins and regulate the expression of functional genes by competitively binding miRNAs with functional genes during transcription^33^. In addition, it can also produce endogenous small interfering RNAs to inhibit the expression of functional genes^34^. This implies that the pseudogene may gain new biological roles while losing the original gene function. In this study, it was found that *P. cheilanthifolia* has a shorter nucleotide sequence but encodes a greater variety of PCGs and that the pseudogenization of the *rps19* gene promotes the cp genome to encode a greater number of PCGs and increases variability (Pi = 0.15671). Understanding this issue can help us grasp the evolutionary process of the genomic compositions in *P. cheilanthifolia,* thus enabling accurate speculation on population changes and evolutionary processes.

The amino acid preference for codons is almost universal in the cp genomes of most higher plants, due to codon parsimony and amino acids' efforts to prevent and overcome transcription errors^26^. During protein synthesis, amino acids show a one to many relationship with codons, except for Met (AUG) and Trp (UGG), which to some extent inhibits genetic mutations from harming the plant body during the evolutionary process, as well as representing the regularity of a particular amino acid^35^. Of course, RSCU plants vary in species and are affected by a variety of factors, such as nucleotide length, the hydrophilic expression level of codons, and base structure^36,37^. In the two species reported in this study, the authors found that their preferred codons for each gene were essentially the same, and the high preference for Leu (UUA), Ala (GCU), and Tyr (UAU), among others, did not differ from that of other *Pedicularis* plants. Simple sequence repeats composed of short (1–6 bp) repeated nucleotide sequences make an important contribution to plant phylogeny, species identification, and taxonomic studies^38^. The results show that *Pedicularis* species have a high A+T bias and very few hexanucleotide repeats, which may be caused by the high number of AT and TA dinucleotide repeats produced by plants subjected to environmental influences.

The large variation in the nucleotide diversity values (0 < Pi < 0.15671) of the 10 *Pedicularis* species implies that the nucleotide substitutions in *Pedicularis* plants had a higher level of diversity during evolution. In this study, it was found that the Pi values of the IR region of the cp genome of the investigated *Pedicularis* species were much lower than those of the LSC and SSC regions, and the SSRs were also more abundant in the LSC and SSC regions. Multiple pieces of evidence suggest that the IR region is conserved because its nucleotide sequences have the properties of inverted repeats; however, LSC and SSC are high-mutation regions, enriching the species diversity of *Pedicularis*. In this study, several highly variable genes (*rpl22*, *rps19*, *rpl12*, *ycf1*, *trnH*, *psbA*, and *ndhH*) and several highly variable regions (*trnS*-*GGA*, *trnV*-*UAC*, *ndhJ*-*trnV*, *ycf4*-*cemA*, *ndhE*-*nhdG*, and *rpl32*-*trnL*) were investigated. In terms of boundary genes, the *ycf1* gene was affected by the expansion of the IR region, and only a small portion of the sequence (3–106 bp) crossed the SSC/IRb boundary; for most of the nucleotide sequence crossing the SSC/IRa boundary into the SSC region, however, 4, 322-4, 907 bp of the nucleotide sequence remaining in the IRa region was identified. The *trnH* gene was very active at the IRa/SSC boundary, located within the LSC region, but it showed a tendency to move closer to the Ira region. In recent years, studies on plant cp genomes and mitochondrial genomes have pointed out that different levels of base exchange between cp genomes and mitochondrial genomes occurred during the evolution of plants^39^, and the *trnH* gene is thought to be possibly involved in this process^40^, so the *trnH* gene probably moved away from the IRa region near the LSC region to facilitate more gene exchange with the mitochondrial genome^41^.

As an independent monophyletic genetic unit, the cp genome can also encode genes related to its own function. It does not undergo recombination during the evolution of plants and can directly reflect the genetic variation accumulated in the long-term evolution of plants^42^. The cp genome contains a large number of repeat sequences, which is an important basis for studying the evolutionary process and genetic characteristics of species^43^. Chloroplast genome is suitable for studying the interspecific and intraspecific genetic diversity of related species. The genetic relationship between related species is far and near, and it can also be used to trace the origin and migration of species^44^. However, due to natural selection and environmental pressure, *Pedicularis* is experiencing large interspecific variation, and the complete cp genome can provide important data support for its taxonomic research.The results of this study showed that *P. kansuensis* and *P. olivriana*, *P. chinensis* and *P. aschistorhyncha* were sisters. However, due to natural selection and environmental pressures, the chloroplast genome of *Pedicularis* plants is undergoing large interspecific variation, showing biodiversity. The order of Pi-based variation is SSC > LSC > IR, and the coding region is far more conservative than the non-coding region. Therefore, the ycf1 gene and three non-coding regions of *trnV*-*UAC*, *ndhJ*-*trnV* and *ycf4*-*cemA* in the SSC region have universal reference value for the development of DNA barcoding or play a role in the future identification of *Pedicularis* plants. Among them, the high mutation of *ycf4*-*cemA* has been proved and widely used in the study of geographical pedigree^45^. In conclusion, more chloroplast genome data will promote the classification of *Pedicularis* plants.

## Materials and methods

### Plant material, DNA extraction, cp genome sequencing, and assembly

In June 2022, specimens of *P. chinensis* were collected in Hualong County, Haidong City, Qinghai province (N36° 13′ 26′′, E102° 19′ 50′′, Altitude: 3, 839 m); specimens of *P. kansuensis* were collected in Hualong County, Haidong City, Qinghai province (N36° 11′ 21′′, E102° 18′ 42′′, Altitude: 3, 896 m). The morphological identification of both studied species was performed by Professor Yuling Li (1991990033@qhu.edu.cn) of Qinghai University at the time of field collection. Research material was sealed in centrifuge tubes, preserved in liquid nitrogen, and stored in the Herbarium of Cordyceps Research Unit, College of Animal Husbandry and Veterinary Sciences, Qinghai University, under the designations MXH-zg13 and MXH-gs14. No materials were sourced from specially protected areas, and local collection permits and ethical guideline approval were obtained.

Total DNA was extracted using a plant genomic DNA extraction kit (Merck Co. Ltd; Beijing, China, https://www.sigmaaldrich.cn/CN/zh/applications/genomics); DNA quality was verified via 1.0% agarose gel electrophoresis, and DNA concentration was measured via Qubit 3.0 (Thermo Fisher Scientific, USA) fluorescence. The total genomic DNA was assayed, an insert library of approximately 350 bp in length was constructed, and double-end sequencing with a read length of 150 bp was performed using an Illumina HiSeq X Ten platform^46^. An NGS QC Tool Kit was used for checking the quality of the raw reads (default parameters), filtering and removing the adapters and low-quality reads, and obtaining high-quality reads for analysis. The whole cp genome sequence of *P. cephalantha* (accession number: NC060560) was used as the seed of the cp genome extension seed for this study, and AFRESh software was used to screen, compare, and assemble the clean reads to obtain sequence overlap cluster contigs^47^. The contigs were spliced into loops, and redundant sequences were removed using Bandage software^48^; the splicing results were compared with the above-seeded sequences in Geneious software to determine the orientation of inverted repeat (IR) regions^49^, which was used to initially assemble the whole cp genome sequences from scratch. The original sequences were then remapped onto the assembled cp whole-genome sequence using Bowtie2, and the boundaries between the large single copy (LSC) region, the small single copy (SSC) region, two inverted repeat regions, and the splicing of each contig were evaluated and verified by detecting the sequence coverage^50^. The complete cp genome sequences were edited using Sequin software and submitted to GenBank under the accession numbers OQ587613 and OQ587614 for *P. kansuensis* and *P. chinensis*, respectively.

### Characterize genome annotation and characterization

The cp genome was annotated using GeSeq software^51^, and the annotation results were manually corrected using Geneious Prime^52^. The boundaries of all tRNA genes were determined using the online software tRNAscan-SE (http://lowelab.ucsc.edu/tRNAscan-SE/)^53^. Chloroplast genome mapping was performed online using Organellar Genome DRAW (OGDRAW) software (https://chlorobox.mpimp-golm.mpg.de/OGDraw.html)^54^.

### Analysis of repetitive sequences and codon preference

REPuter software (https://bibiserv.cebitec.uni-bielefeld.de/reputer) was used to detect dispersed repeats in the cp genome sequences with the following parameters set: minimum repeat length of 30 bp and a Hamming distance of 3 (> 90% similarity between repeats)^55^. Tandem repeats were detected using Tandem Repeats finder software (https://tandem.bu.edu/trf/trf.html)^56^, with default values for parameters. Information on the cp genomes was entered into MISA software^57^, and simple repetitive sequences in the cp genomes of all species were detected separately with the parameters of single-nucleotide units, dinucleotide, trinucleotide, tetranucleotide, pentanucleotide, and hexanucleotide repeats set to 10, 5, 4, 3, 3, and 3, respectively, and the types, numbers, and distribution patterns of SSRs were analyzed.

### Analysis of contraction and expansion of IR region

One of the most important sources of cp genome size variation is IR/SSC boundary changes^58^. mVISTA software was used for a comparative analysis of the complete cp genomes of *P. chinensis* and *P. kansuensis* and the plastomes of 8 other representatives of the genus *Pedicularis* under the LAGAN model^59^. An LSC/IR/SSC boundary comparison of the cp genomes of the 10 plant species was performed using IRscope software (https://irscope.shinyapps.io/irapp/) to identify possible contractions and expansions of the IR regions in *Pedicularis*^60^.

### Phylogenetic analysis *Pedicularis*

To accurately determine the taxonomic position of *P. chinensis* and *P. kansuensis*, the cp genome sequences of 66 species representing the family Orobanchaceae were collected from NCBI for a phylogenetic analysis (Table S2). Species were selected as follows: 25 genera in the family Orobanchaceae, with at least 1 species per genus, increasing the number of species selected for species-rich genera. *Scrophularia henryi* of the genus *Scrophularia* in the family Scrophulariaceae was used as an outgroup. Mega software was used to compare the complete cp genome data of the above 66 species. Based on the obtained 66 nucleotide sequences, the phylogenetic tree was constructed by maximum likelihood (ML) method, and the parameters were set as Models = GTR + GAMMA, Bootstrap = 1 000^61,62^.

### Ethical approval and consent to participate

The plant specimen is not an endangered species. It does not require specific permissions or licenses. The collection was carried out in accordance with the guidelines provided by Qinghai University and the relevant requirements of international law.

### Supplementary Information

Supplementary Tables.

## Data Availability

All data generated or analyzed during this study are included in this paper and its supplementary information files, which are also freely available from the corresponding authors. The genome sequence data of *P. kansuensis* and *P. chinensis* that support the findings of this study are openly available in GenBank of NCBI under the accession (https://www.ncbi.nlm.nih.gov/search/all/?term=OQ587613) and (https://www.ncbi.nlm.nih.gov/search/all/?term=OQ587614).
